# Plant Essential Oils Synergize and Antagonize Toxicity of Different Conventional Insecticides against *Myzus persicae* (Hemiptera: Aphididae)

**DOI:** 10.1371/journal.pone.0127774

**Published:** 2015-05-26

**Authors:** Nicoletta Faraone, N. Kirk Hillier, G. Christopher Cutler

**Affiliations:** 1 Department of Environmental Sciences, Faculty of Agriculture, Dalhousie University, Truro, Nova Scotia, B2N 5E3 Canada; 2 Department of Biology, Acadia University, Wolfville, Nova Scotia, B4P 2R6 Canada; University of Crete, GREECE

## Abstract

Plant-derived products can play an important role in pest management programs. Essential oils from *Lavandula angustifolia* (lavender) and *Thymus vulgaris* (thyme) and their main constituents, linalool and thymol, respectively, were evaluated for insecticidal activity and synergistic action in combination with insecticides against green peach aphid, *Myzus persicae* (Sulzer) (Hemiptera: Aphididae). The essential oils and their main constituents exerted similar insecticidal activity when aphids were exposed by direct sprays, but were non-toxic by exposure to treated leaf discs. In synergism experiments, the toxicity of imidacloprid was synergized 16- to 20-fold by *L*. *angustifolia* and *T*. *vulgaris* essential oils, but far less synergism occurred with linalool and thymol, indicating that secondary constituents of the oils were probably responsible for the observed synergism. In contrast to results with imidacloprid, the insecticidal activity of spirotetramat was antagonized by *L*. *angustifolia* and *T*. *vulgaris* essential oils, and linalool and thymol. Our results demonstrate the potential of plant essential oils as synergists of insecticides, but show that antagonistic action against certain insecticides may occur.

## Introduction

Synthetic chemical insecticides have been instrumental in the evolution of modern agriculture. Nevertheless, overuse and misuse of pesticides has sometimes resulted in problems of environmental contamination, poisoning, pesticide resistance, and pest resurgence. These issues, consumer demands for low-risk products, and legislative withdrawal of older chemistries in many jurisdictions has resulted in increased attention towards reduced-risk tactics for pest management.

“Biopesticides” are reduced-risk pesticides usually classified as formulated microbial pathogens or plant derived biochemicals with activity against pest species [1]. An increasingly studied subset of biopesticides are plant essential oils (EOs) [2,3]. EOs are volatile, natural compounds formed by aromatic plants as secondary metabolites that may serve to protect plants from herbivores or pathogens [4,5]. The oils, which are generally composed of complex mixtures of monoterpenes, phenols, and sesquiterpenes, have demonstrated insecticidal, repellent, antifeedant and insect growth regulatory properties, and suppression of adult insect fertility and oviposition [4,6]. EOs have proven effective in plant protection against plant chewing and sucking insect pests, as well as flies that oviposit in fruit [4].

Several practical, environmental, and biochemical characteristics of EOs make them a potential alternative for insect pest management [3,4]. However, EOs have seen limited use in crop protection. Key among these limitations are inconsistencies in efficacy and composition, lower potency against target pests relative to many synthetic insecticides, and relatively lower persistence and residual activity. The latter three in particular may restrict the use of EOs as stand-alone products for crop protection in many situations [6].

It has previously been suggested that EOs might be used in alternation with synthetic insecticides to manage insect resistance problems [6]. An alternative strategy for the use of EOs in crop protection could be to use them in mixtures with conventional insecticides. The idea is intriguing because it may increase opportunities for use of EOs in pest management, while reducing inputs of synthetic insecticides. This might be especially true if certain EOs were to synergize the activity of synthetic insecticides. Synergists are substances that when given in sublethal amounts increase the potency of an insecticide, mainly by inhibiting cytochrome P450 monooxygenases and other enzyme systems that metabolize insecticide molecules [7,8]. Piperonyl butoxide, sulfoxide, and sesamex are common synthetic synergists, but it has been suggested that plants naturally possess and utilize synergists in order to overcome injury from phytophages [7]. A limited number of investigations have shown that EOs in combination or when mixed with other botanical insecticides can have synergistic insecticidal activity against Lepidoptera and Diptera [9–14]. There have been fewer examinations into combined use of EOs with insecticides, although synergistic activity of mixtures of EOs + insecticides have been reported against field crop pests [3], stored product pests [15], and mosquitos [16].

In the current study, we tested the hypothesis that EOs could synergize the activity of two common synthetic insecticides against green peach aphid, *Myzus persicae* (Sulzer) (Hemiptera: Aphididae). This aphid is an agricultural pest of global economic importance that is managed mainly with synthetic insecticides [17–19]. We focused on readily available EOs from lavender, *Lavandula angustifolia* Mill. (Lamiaceae), and thyme, *Thymus vulgaris* L. (Lamiaceae), and two of their main constituents, linalool and thymol, respectively. We tested these in combination with imidacloprid and spirotetramat, two insecticides that are effective against *M*. *persicae*, but have different modes of action [20–22].

## Materials and Methods

### Plant and insect maintenance

Cabbage plants, *Brassica oleracea capitata* L. (cv Copenhagen market) were grown in a greenhouse in 100 mm diameter pots containing Pro-Mix potting soil. Soluble fertilizer was given at planting, and plants were watered as needed. Foliage used for insect rearing or experiments was from plants 4–6 weeks old. Cabbage foliage was used for all laboratory bioassays.

*M*. *persicae* was originally collected from a wild population infesting cabbage plants in the Environmental Sciences greenhouses on the Dalhousie University Agricultural Campus. Collected aphids were reared on cabbage foliage in 6 L plastic boxes (37 L × 24 W × 14 H cm) lined with moistened paper towel. Boxes were held in a growth chamber (22±2°C, 16:8 L:D, 65±5% RH) and cabbage foliage was changed every 2–3 days. Before experiments, groups of 50 adult aphids were transferred to plastic boxes with cabbage foliage. After 24 h, all adults were transferred to new boxes so that old boxes contained cohorts of nymphs of the same age. Second instar nymphs were used at the start of bioassays.

### Chemicals

Imidacloprid (240 g L^-1^; Admire 240F, Bayer CropScience Canada Inc., Calgary, AB), a neonicotinoid insecticide, and spirotetramat (240 g L^-1^; Movento 240, Bayer CropScience Canada Inc., Calgary, AB), a tetramic acid derivative (ketoenole) insecticide, were used in experiments. EOs from *L*. *angustifolia* and *T*. *vulgaris* were purchased from Golden Bough Botanical Inc. (Delta, BC, Canada), and linalool (97% purity) and thymol (98% purity) were purchased from Alfa Aesar Inc. (Ward Hill, MA, USA). Serial dilutions of imidacloprid (0.03–1.0 mg L^-1^), spirotetramat (0.3–30 mg L^-1^), EOs, and linalool (0.3E4–10E4 mg L^-1^) were prepared in distilled water with 0.1% v/v Tween-80 [23]. Because thymol is a crystalline solid that is more difficult to dissolve into water, thymol serial dilutions (0.3E4–10E4 mg L^-1^) were prepared in distilled water with 0.5% v/v Tween-80 and 1% v/v acetone.

### Essential oil composition

EO analysis was carried out using a Bruker Scion 456 TQ GC-MS/MS system (Bruker Ltd., Milton, ON, Canada) equipped with an auto-sampler (CombiPAL Auto-sampler control, Bruker Ltd.). Separations were carried out using a Bruker capillary column BR-5ms (15 m × 0.25 mm I.D. × 0.25 μm film thickness (df)). The GC oven temperature was programmed to 50°C for 0.5 minutes, followed by gradual increments of 5°C/min until it reached 150°C, where it was held for 2 minutes, followed by gradual increments of 30°C/min until reached 250°C, where it was held for 1.2 minutes. The total analysis time was 25 minutes. Samples were injected using an auto-sampler injector with a split ratio of 1:20. The injector temperature was 250°C. Helium was used as the carrier gas at a flow rate of 2 mL/min. The MS was operated at an electron ionization (EI) energy of 70 eV with the ion source temperature at 250°C. The mass scan range was m/z 35–350. Compounds were identified by a combination of retention indices and mass spectra found within libraries and standards [24,25]. Quantification of the EO constituents (expressed as percentages) was carried out by peak area normalization. The identification was based on the comparison of mass spectra and retention indexes with published results [26] and by injection of standard solutions.

### Insecticidal activity

For direct contact exposure, cohorts of five insects were placed in clean glass Petri plates (9 cm diameter) and sprayed in a Potter tower (Burkard Scientific, Uxbridge, UK) at 78 kPa with 2 mL of imidacloprid, spirotetramat, *L*. *angustifolia* EO, *T*. *vulgaris* EO, linalool, or thymol solution. For each test compound, a range of concentrations was chosen as described above that resulted in approximately 10–90% mortality, as determined from preliminary tests. After exposure in the Potter tower, treated insects were transferred using a fine paintbrush to plastic Petri plates (5.5 cm diameter) lined with Whatman No. 1 filter paper containing one untreated 1.5 cm diameter cabbage leaf disc.

In another set of experiments, aphids were exposed to treated leaf discs. Cabbage leaf discs were individually sprayed in the Potter tower with 2 mL of test solution as described above. After exposure, treated leaf discs were left to dry for 10–15 minutes and transferred individually to plastic Petri plates (5.5 cm diameter) lined with Whatman No. 1 filter paper. Five untreated aphid nymphs were placed on each treated leaf disc. Petri dishes were placed in a covered plastic box (37 L x 24 W x 14 H cm) lined with moist paper towels, and placed in a growth chamber (25±2°C, 16:8 L:D, 65±5% RH). Mortality was determined under a dissecting microscope after 24 h, except for experiments with slower acting spirotetramat, for which mortality was assessed after 48 h.

In both direct contact and leaf disc exposures, experiments were done as a randomized complete block design. Each bioassay had at least five concentrations (one control and 4–5 treatments), and for each there were three Petri dishes with five aphids per dish. Each bioassay was considered an experimental block, and bioassays were conducted three times for a total of 45 insects per treatment. Fresh solutions were prepared for every experimental block.

### Synergism experiments

Bioassays were done to determine if lavender and thyme EOs, or their main constituents, linalool and thymol, would synergize the activity of imidacloprid and spirotetramat against *M*. *persicae*. Insects were exposed by direct spray or through exposure to treated leaf discs, as described above. In bioassays with synergists, the dose/concentration of synergist used should be a maximum dose that causes no observable toxicity [27]. From the previously described experiments, we determined that the direct exposure no observable effects concentration (NOEC) of lavender and thyme EOs was approximately 0.3% v/v. The NOEC for linalool and thymol was approximately 1% v/v and 2% w/v, respectively. These concentrations were used in direct spray exposure synergism experiments (Table 1). No NOEC for EOs, linalool, and thymol could be established for treated leaf exposure, so concentrations the same as those in direct spray synergism experiments were used in leaf disc exposure synergism experiments. The only exception was leaf disc synergism experiments with thymol, where 0.5% w/v thymol concentration was used, as concentrations higher than this were phytotoxic (Table 1). Insects or leaf discs were exposed to the insecticide + EO/linalool/thymol mixtures in a Potter tower with a range of concentrations that caused approximately 10–90% mortality, as described above. Mortality was recorded at 24 h or 48 h. Experiments were a randomized complete block design, with five insects per Petri dish and three replicate Petri dishes of insects per treatment. Bioassays were conducted three times, each constituting an experimental block.

**Table 1:** Exposure treatments used to test the ability of lavender essential oil (EO), thyme EO, linalool, or thymol to synergize the insecticidal activity of imidacloprid and spirotetramat against *Myzus persicae*.

Synergist	Exposure	Solvent
Lavender EO (0.3% v/v)	Direct and treated leaf disc	H_2_O + 0.1% v/v Tween-80
Thyme EO (0.3% v/v)	Direct and treated leaf disc	H_2_O + 0.1% v/v Tween-80
Linalool (1.0% v/v)	Direct and treated leaf disc	H_2_O + 0.1% v/v Tween-80
Thymol (2.0% w/v)	Direct	H_2_O + 0.5% v/v Tween-80 + 1% v/v acetone
Thymol (0.5% w/v)^a^	Treated leaf disc	H_2_O + 0.5% v/v Tween-80 + 1% v/v acetone

^a^ Thymol concentrations above 0.5% w/v were phytotoxic.

### Data analysis

Proc Probit [28] was used to calculate median lethal concentrations (LC50) at 24 or 48 h for the two insecticides, EOs, linalool, and thymol. Synergism ratios (LC50 of insecticide alone relative to the LC50 of insecticide in combination with the EO) [27] and ratio tests [29] were used to compare LC50 values of insecticides alone to LC50 values of insecticides when combined with EOs or their main constituents.

## Results

Eight EO compounds isolated from *L*. *angustifolia* were detected and quantifiable, >92% of which consisted of linalool and linalyl acetate. The remaining detectable constituents in the *L*. *angustifolia* EO consisted mostly of camphor and various monoterpenes (Table 2). Thirteen compounds were identified in *T*. *vulgaris* EO, which was dominated by thymol (>72%). Linalool (2.4%) and various terpenes comprised the remaining 15% of identifiable constituents of *T*. *vulgaris* EO (Table 2).

**Table 2:** Composition of essential oils extracted from Lavandula angustifolia and *Thymus vulgaris*.

Compound	RT	*L*. *angustifolia* (%)	*T*. *vulgaris* (%)
α-Pinene	2.376	0.19	6.44
Camphene	2.571	-	0.15
β-Pinene	2.993	0.05	1.3
3-Carene	3.438	0.04	1.58
α-Terpinene	3.677	-	0.18
*p*-Cymene	3.812	0.48	0.06
*R*-Limonene	3.901	-	2.51
γ-Terpinene	4.481	-	0.1
Terpinolene	5.054	-	0.09
Linalool	5.436	54.29	2.41
Camphor	6.347	2.17	-
Linalyl acetate	9.092	38.56	0.09
Thymol	10.263	-	72.68
Geranyl acetate	12.275	0.02	-
Methyl-Jasmonate	18.201		0.002
other^a^	-	4.2	12.4

^**a**^ unidentified compounds

In all bioassays control mortality was less than 8%, and the probit model adequately described all dose responses for EOs and insecticides alone and in combination with EOs or their main constituents (Tables 3–5). By direct contact, imidacloprid and spirotetramat were highly toxic to aphids, and over four orders of magnitude more potent than the EOs, linalool, and thymol (Table 3). The slopes of the probit lines of linalool and thymol were noticeably greater than those of the insecticides and EOs. Imidacloprid and spirotetramat were also highly toxic to aphids when exposed to treated foliage, although spirotetramat was the more potent insecticide through this exposure route (Table 3). No aphid mortality occurred following exposure to foliage treated with EOs, linalool, or thymol; tests with EOs on foliage were done up to concentrations of 3.0E4 ppm, above which phototoxicity was observed (Table 3). We did observe that in some cases aphids were repelled from the treated leaf discs, often being seen on the filter paper instead of the discs, but aphids were most often observed on the treated foliage.

**Table 3:** Susceptibility^a^ of *Myzus persicae* second instar nymphs to direct sprays or treated leaf discs of imidacloprid, spirotetramat, lavender (*Lavandula angustifolia*) and thyme (*Thymus vulgaris*) essential oils, linalool, and thymol.

Treatment	Exposure route	LC50 (ppm) (95%CL)	Slope (±SEM)	*χ* ^*2*^	*df*	*P*
Imidacloprid	Direct	0.79 (0.59–1.07)	1.73 (0.22)	3.11	2	0.21
	Leaf disc	1.30 (0.89–1.83)	1.38 (0.20)	2.26	2	0.32
Spirotetramat	Direct	1.61 (0.26–7.57)	0.42 (0.17)	1.44	2	0.49
	Leaf disc	0.12 (0.04–0.4)	0.35 (0.06)	5.35	3	0.15
*L*. *angustifolia*	Direct	5.06E4 (2.84E4–9.52E4)	0.95 (0.18)	7.33	3	0.06
	Leaf disc^c^	---	---	---	---	---
*T*. *vulgaris*	Direct	5.27E4 (2.95E4–10.3E4)	0.93 (0.18)	7.38	3	0.06
	Leaf disc^c^	---	---	---	---	---
Linalool	Direct	4.85E4 (4.36E4–5.35E4)	7.54 (1.03)	0.61	2	0.74
	Leaf disc^c^	---	---	---	---	---
Thymol^b^	Direct	7.04E4 (6.73E4–7.36E4)	16.4 (2.26)	0.40	2	0.53
	Leaf disc^c^	---	---	---	---	---

^a^ All 24 h exposure except spirotetramat, which was 48 h exposure.

^b^ A different solvent was needed to prepare thymol solutions. See Table 1 for details.

^c^ No significant mortality of aphids exposed to treated leaf discs at any concentration that was not phytotoxic.

**Table 4:** Susceptibility of *Myzus persicae* second instar nymphs to direct sprays or treated leaf discs of imidacloprid mixed with essential oils of *Lavandula angustifolia* and *Thymus vulgaris*, linalool or thymol.

Treatment		Exposure route	LC50 (ppm) (95%CL)	Slope (±SEM)	*χ* ^*2*^	*df*	*P*	Synergism ratio^a^	Z value, *P* ^b^
Imidacloprid		Direct	0.79 (0.59–1.07)	1.73 (0.22)	3.11	2	0.21	---	---
		Leaf disc	1.30 (0.89–1.83)	1.38 (0.20)	2.26	2	0.32	---	---
	+ *L*. *angustifolia*	Direct	0.05 (0.02–0.08)	1.11 (0.20)	3.20	2	0.20	15.8	4.32, <0.001
		Leaf disc	0.72 (0.45–1.30)	0.90 (0.18)	3.21	2	0.20	1.8	0.58, 0.56
	+ *T*. *vulgaris*	Direct	0.04 (0.02–0.06)	1.25 (0.22)	2.24	2	0.32	19.8	4.78, <0.001
		Leaf disc	0.44 (0.28–0.67)	1.09 (0.19)	4.48	2	0.11	3.0	1.51, 0.13
	+ Linalool	Direct	0.28 (0.21–0.38)	2.75 (0.31)	4.88	3	0.30	2.8	1.57, 0.12
		Leaf disc	0.72 (0.45–1.24)	0.99 (0.19)	1.53	2	0.47	1.8	0.61, 0.54
Imidacloprid^c^		Direct	0.45 (0.07–2.35)	1.78 (0.35)	4.66	2	0.10	---	---
		Leaf disc	0.92 (0.51–1.42)	1.02 (0.18)	3.48	2	0.18	---	---
	+ Thymol^c^	Direct	0.25 (0.19–0.35)	2.64 (0.33)	5.98	3	0.11	1.8	1.69, 0.091
		Leaf disc	0.19 (0.12–0.28)	1.20 (0.19)	0.14	2	0.93	4.8	0.57, 0.57

^a^ LC50 of imidacloprid divided by LC50 of imidacloprid+EO (or main component), for each exposure route.

^b^ Ratio test [29] comparing the LC50 of imidacloprid to the LC50 of imidacloprid+EO (or main component), for each exposure route.

^c^ A different solvent was needed to dissolve thymol, thus necessitating a separate LC50 determination for imidacloprid. See Table 1 for details of solvent and synergist concentrations.

**Table 5:** Susceptibility of *Myzus persicae* second instar nymphs to direct sprays or treated leaf discs of spirotetramat mixed with essential oils of *Lavandula angustifolia* and *Thymus vulgaris*, linalool or thymol.

Treatment		Exposure route	LC50 (ppm) (95%CL)	Slope (±SEM)	*χ* ^*2*^	*df*	*P*	Synergism ratio^a^	Z value, *P* ^b^
Spirotetramat		Direct	5.07 (3.29–7.74)	1.12 (0.19)	1.25	2	0.53	---	---
		Leaf disc	0.17 (0.05–0.48)	0.43 (0.09)	3.63	3	0.16	---	---
	+ *L*. *angustifolia*	Direct	17.26 (10.56–38.53)	0.99 (0.19)	0.74	2	0.69	0.3	7.48, <0.001
		Leaf disc	0.96 (0.64–1.62)	1.10 (0.19)	4.39	2	0.11	0.2	0.29, 0.77
	+ *T*. *vulgaris*	Direct	10.57 (6.15–23.78)	0.81 (0.18)	2.21	2	0.33	0.5	4.07, <0.001
		Leaf disc	0.55 (0.38–0.80)	1.28 (0.20)	1.78	2	0.41	0.3	2.74, 0.0062
	+ Linalool	Direct	17.97 (10.50–45.93)	0.91 (0.19)	1.57	2	0.46	0.3	7.38, <0.001
		Leaf disc	0.17 (0.01–6.72)	0.17 (0.77)	4.67	3	0.20	1.0	0, 0.99
Spirotetramat^c^		Direct	2.06 (1.33–3.30)	1.06 (0.19)	0.95	2	0.62	---	---
		Leaf disc	0.10 (0.06–0.14)	1.34 (0.20)	1.08	2	0.58	---	---
	+ Thymol^c^	Direct	4.74 (2.97–9.36)	1.02 (0.19)	2.36	2	0.31	0.4	2.39, 0.017
		Leaf disc	0.10 (0.05–0.15)	0.99 (0.18)	0.94	2	0.62	1.0	0, 0.99

^a^ LC50 of spirotetramat divided by LC50 of spirotetramat+EO (or main component), for each exposure route.

^b^ Ratio test [29] comparing the LC50 of spirotetramat to the LC50 of spirotetramat+EO (or main component), for each exposure route.

^c^ A different solvent was used to dissolve thymol, thus necessitating a separate LC50 determination for spirotetramat. See Table 1 for details of solvent and synergist concentrations.

In synergism experiments, aphids were 16- to 20-fold more susceptible to direct sprays of imidacloprid when this insecticide was mixed with low concentrations of *L*. *angustifolia* or *T*. *vulgaris* EOs (Table 4). However, exposure to foliage treated with a mixture of imidacloprid and EO resulted in only 2- to 3-fold increased susceptibility compared to exposure to foliage treated only with imidacloprid. The addition of NOEC concentrations of linalool or thymol to imidacloprid gave synergism ratios of 1.8–4.8 depending on the exposure route, but this did not result in significant changes in LC50 values (Table 4).

Unlike the synergism observed in some imidacloprid bioassays, in several cases the insecticidal activity of spirotetramat was antagonized when mixed with EOs. NOEC concentrations of *L*. *angustifolia* EO, *T*. *vulgaris* EO, linalool, and thymol antagonized the activity of spirotetramat against aphids by 2- to 3-fold through direct contact exposure. Leaf disc exposures of aphids to mixtures of spirotetramat with these compounds did not result in significant changes in LC50 values compared to leaf disc exposure to spirotetramat alone (Table 5).

## Discussion

We demonstrated that low amounts of readily available EOs can result in significant synergism of a widely used insecticide. This result is significant given that although insecticidal activity of many EOs has been reported [4], their widespread application in pest management has been limited, largely due to inferior performance relative to synthetic insecticides [6,30]. At the same time there has been a push to reduce inputs of conventional insecticides. The synergism of imidacloprid by both *L*. *angustifolia* and *T*. *vulgaris* points to a potential solution to both issues as it increases opportunities to use EOs in pest management while reducing the amount of synthetic insecticide needed to suppress pest populations. On the other hand, results of our experiments with spirotetramat highlight the fact that plant EOs may also antagonize the bioactivity of insecticides and suggest that insecticide mode of action or chemical/physical properties are an important determinate of whether synergism or antagonism of insecticide-EO combinations will occur.

We found that lavender and thyme EOs, linalool, and thymol were toxic to aphids, albeit at concentrations several orders of magnitude greater than lethal concentrations of imidacloprid and spirotetramat. The slopes of the regression lines for linalool and thymol were very steep, indicating a much more homogenous toxic response among aphids to these compounds relative to that for imidacloprid, spirotetramat, and the EOs. Insecticidal and behavior modifying activity of lavender and thyme EOs has previously been shown against aphids [31–35]. We found that *L*. *angustifolia* EO contained high amounts of linalool, as others have reported [36]. Linalool can inhibit acetyl cholinesterase [37], which probably, at least in part, accounts for the insecticidal effects of lavender EO. Our *T*. *vulgaris* EO contained high amounts of thymol, a monoterpene phenol that binds to post-synaptic GABA receptors associated with chloride channels in insects [38]. Thymol insecticidal activity has also been linked to interference with the tyramine receptor cascade, which leads to synthesis of octopamine, an important neurotransmitter, neurohormone, and neuromodulator in insects [39]. When directly applied to aphids, the LC50 values of *L*. *angustifolia* and *T*. *vulgaris* were very similar to those of linalool and thymol, which suggested that the insecticidal activity of the EOs was mainly due to their primary constituents. EOs, linalool, and thymol were non-toxic to aphids in leaf disc treatment bioassays. Although aphids did feed on the treated foliage, they were often observed off the foliage, suggesting a repellency effect resulted in reduced exposure and bioactivity.

In experiments where aphids were exposed to a combination of insecticide with EO or an EO constituent, when synergism occurred it was much greater through direct exposure bioassays than leaf treatment bioassays. This was possibly due in part to a repellent effect of treated foliage to the aphids, as discussed above. There have been a few other reports of EOs or their constituents synergizing other synthetic or botanical insecticides. Terpinen-4-ol and *γ*-terpinene, the main constituents of *Majorana hortensis* (Lamiaceae), increased the insecticidal activity of the synthetic insecticides profenofos and methomyl by 2- to 3-fold against *Spodoptera littoralis* (Lepidoptera: Noctuidae) larvae and *Aphis fabae* (Hemiptera: Aphididae) adults [40]. In other experiments with *S*. *littoralis*, trans-anethole, which is derived from anise (*Pimpinella anisum*: Apiaceae), synergized thymol, citronellal, and R-terpineol in both acute toxicity and feeding deterrence experiments [12]. Botanical extracts of *Khaya senegalensis* (Meliaceae), *Daucus carota* (Apiaceae), or *Callitris glaucophylla* (Cupressaceae) synergized the activity of fenitrothion or lambda-cyhalothrin against *Culex annulirostris* (Diptera: Culicidae) larvae [41], and extracts of *Piper nigrum* (Piperaceae) synergized the botanical insecticide pyrethrum against *Drosophila melanogaster* (Diptera: Drosophilae) [9]. Importantly, there is evidence in the field that mixtures of EOs and conventional insecticides can effectively control pests like thrips and aphids [3].

Most interesting in our synergism experiments was that: (1) the EOs induced far more synergism than linalool or thymol; and (2) EOs synergized imidacloprid but antagonized spirotetramat. The greater (3- to 9-fold) synergism with EOs relative to linalool and thymol was somewhat unexpected given that when applied alone, most of the insecticidal activity against aphids seemed to be due to these primary constituents. This indicates that secondary constituents in the EOs were needed to elicit synergism. It is hypothesized that because plants usually defend themselves against herbivores using a suite of compounds rather than individual ones, minor or secondary constituents of EOs that are found in low percentages may be synergists that enhance the effectiveness of the major constituents [12,42–44]. Certain minor constituents were found in both lavender and thyme EOs, and it is possible that one of these accounted for the synergism of both EOs. Alternatively, a unique combination of secondary constituents in each EO may have caused the synergism. As previously suggested, identifying the key synergistic compounds within complex EO mixtures could allow for the development of highly effective control agents [12].

Insecticide synergists are considered natural or synthetic chemicals that are non-toxic at a tested concentration/dose but increase the lethality of an insecticidal compound [7,45]. Non-toxic concentrations of both lavender and thyme EOs synergized the direct contact activity of imidacloprid in our experiments. It is possible that the observed synergism was the result of the insect being overwhelmed by the mixture of two chemicals that attack different target sites; imidacloprid is an agonist of post-synaptic nicotinic acetylcholine receptors [21], whereas lavender EO inhibits acetyl cholinesterase [37], and thyme EO interferes with post-synaptic GABA receptors and octopamine synthesis [39]. However, when examining the joint action of insecticides, true synergism from a mixture of two or more insecticidal compounds is rarely found [45]. Synergistic action is usually attributed to inhibition of enzyme systems important in the metabolism of insecticides. Such activity has been reported in EOs from sesame, *Amyris*, sandalwood, *Helichrysum*, cedar wood, and black pepper that exhibited synergistic effects in *Aedes aegypti* (Diptera: Culicidae) larvae while inhibiting cytochrome P450 monooxygenases and/or carboxylesterases *in vitro* [16]. Reduced esterase, glutathione-S-transferase, and/or monooxygenase activity following exposure to various EOs was similarly reported in other pests [46–49], and probably accounts for synergism of imidacloprid in several of our bioassays. This is significant given reports of *M*. *persicae* resistance to imidacloprid, which is primarily though cytochrome P450-mediated detoxification [50].

In contrast to the effects seen with imidacloprid, the insecticidal activity of spirotetramat, an ketoenole insecticide inhibitor of insect lipid biosynthesis [22], was antagonized by EOs, linalool, and thymol in direct contact exposure bioassays. This suggests that synergism vs. antagonism of insecticides with EOs, is contingent on the chemical structure/properties or mode of action of the chemical. Monooxygenases and esterases appear to play a role in insect resistance to ketoenole insecticides [51], but our results of antagonism suggest these were unaffected when spirotetramat was mixed with EOs or primary constituents. Chemical/physical properties of spirotetramat therefore probably accounted for the observed antagonism. Imidacloprid has a much lower log Kow (0.57 at 21°C) and higher water solubility (610 mg/L at 20°C) [52] than spirotetramat (log Kow = 2.51 at pH 7; water solubility = 29.9 mg/L at pH 7 and 20°C) [53], meaning the EOs likely would have been absorbed much more by the lipophilic spirotetramat when mixed in solution. This may have reduced the bioavailability of spirotetramat in aphids. Similarly, Tong and Bloomquist [16] reported that antagonism of permethrin toxicity against mosquito larvae in the presence of EOs was probably due to permethrin’s very high hydrophobicity and affinity with the EOs.

The results of our experiments potentially have valuable implications for integrated pest management. If the laboratory evidence for synergistic effects of thyme and lavender EOs on imidacloprid against *M*. *persicae* applies under greenhouse or field conditions, this could reduce reliance on synthetic insecticides and mitigate potential issues related to environmental contamination, residues on food, non-target impacts, and pesticide resistance. The use of EOs in mixtures as synergistic agents with conventional pesticides may lead to greater uptake of EOs by farmers with opportunities for new crop protection applications and markets. Future work should aim to demonstrate synergism of EOs with synthetic insecticides in the field against aphids and other insect pests, test other EO-insecticide combinations, determine what EO constituents give synergistic activity, and determine the mechanisms of synergism or antagonism.
